# An Extremely Rare Case of Sebaceous Adenoma Involving the Parotid Gland

**DOI:** 10.3390/diagnostics12051232

**Published:** 2022-05-14

**Authors:** Octavian Marius Dincă, Mihai Bogdan Bucur, George Cristian Vlădan, Valentin Nicolae Varlas, Alexandru Bucur

**Affiliations:** 1 Department of Oral and Maxillofacial Surgery, “Carol Davila” University of Medicine and Pharmacy Bucharest, 37 Dionisie Lupu, 020021 Bucharest, Romania; octavian.dinca@umfcd.ro (O.M.D.); alexandru.bucur@umfcd.ro (A.B.); 2 Dentimplant Platinum Aesthetics, 39 Mihail Kogalniceanu, 050103 Bucharest, Romania; mihai_bucur2008@yahoo.com; 3 Department of Obstetrics and Gynecology, “Carol Davila” University of Medicine and Pharmacy Bucharest, 37 Dionisie Lupu, 020021 Bucharest, Romania

**Keywords:** sebaceous tumors, salivary glands tumors, parotid gland

## Abstract

Sebaceous adenoma is an extremely rare tumor located in the parotid gland. In the English literature, less than 10 cases have been reported. Sebaceous adenoma represents 0.5% of all monomorphic adenomas. The authors are presenting a case of sebaceous adenoma of the parotid gland in a 65-year-old female who presented a mass on the left parotid area that had been gradually enlarging for one year without symptoms of pain. On imaging (ultrasound), a well-defined mass lesion in the left parotid area was seen. Histopathological findings were consistent with sebaceous adenoma. Surgical excision is curative. The prognosis is excellent, with a low recurrence rate. The present case report will increase the awareness and possibility of this rare tumor occurring at an unusual site, thereby avoiding any chance of misdiagnosis.

## 1. Introduction

Sebaceous adenoma is a rare benign neoplasm derived from epithelial tissue that displays sebaceous differentiation [1,2] and was first reported by Van Walbeek [3] and was a benign tumor that presents clinically as white-yellowish nodules, usually approximately 5 mm in the largest size [4,5]. On average, sebaceous adenomas occur mainly on the face and scalp [5]. These tumors are most common on the head and neck of persons aged 60 years [4], although sebaceous tumors in the eyelid associated with Muir–Torre syndrome have been found [6]. Extraocular forms are rare—less than 0.1% of all salivary gland neoplasms [7]. Most of these lesions involve the parotid gland, commonly characterized by sebaceous differentiation is common [8]. There have been no reports of recurrence following adequate surgical excision [4].

Herein, we report a case of a 65-year-old woman with sebaceous adenoma of the parotid gland; it is extremely rare insofar as being limited to a few cases reported in the medical literature. After that, we discuss its diagnostic features, including clinical, imaging, and histopathologic findings, with the aim of increasing awareness of this infrequent entity.

## 2. Case Report

A 65-year-old woman presented with a slowly growing palpable mass of the left parotid gland, which was first seen one year earlier. There was no history of pain, bleeding, trismus or discharge from swelling during the course of enlargement, history of fever, or altered salivary flow. She had a 40 years history of smoking. Associated diseases included arterial hypertension and New York Heart Association (NYHA) class III. Physical examination of the left parotid gland disclosed a soft, non-mobile parotid gland tumor of 4 cm in diameter and has no tenderness to palpation. The tumor was covered by a normal-appearing skin not attached to the deeper tissues. The overlying skin appeared with no evidence of scar or discoloration. The skin over the tumor was mobile and pinchable. There was no evidence of cervical lymphadenopathy or neurological affection.

**Figure 1:**
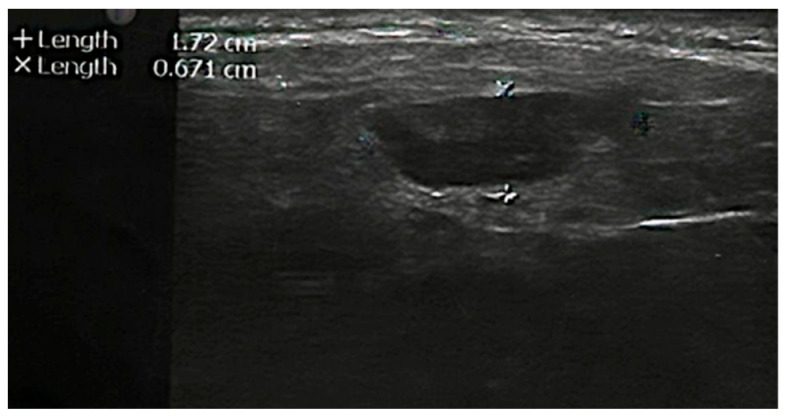
Ultrasonography shows the well-circumscribed tumor in the left parotid.

An ultrasound scan (US) revealed a parotid tumor with well-defined margins, isoechoic texture, and no adenolymphomas (Figure 1). Chest X-rays and laboratory tests were within normal limits.

Based on the history, clinical examination, and ultrasonography findings, a provisional diagnosis of Warthin’s tumor was made. A left superficial parotidectomy with facial nerve preservation was performed. The patient was discharged 7 days after her surgical procedure without any complications.

**Figure 2:**
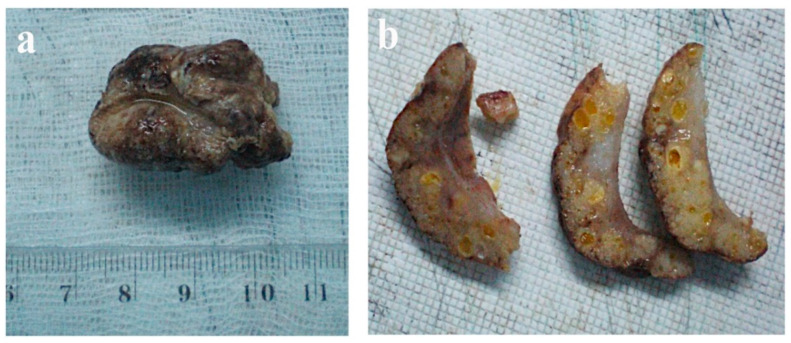
Macroscopic aspects (**a**) tumor after the en-block removal; (**b**) the cut surfaces of the resected tumor.

Histopathology reported a well-circumscribed tumor of 3.2 × 2 × 1.6 cm, surrounded by normal parotid tissue, white, yellowish color, and some cystic spaces. The cut surface was homogeneous, with small cystic spaces (Figure 2a,b).

**Figure 3:**
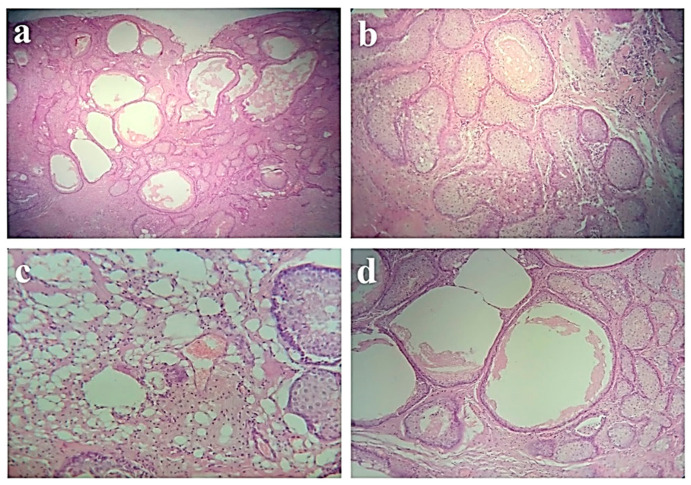
Photomicrographs of the histologic specimen showed (**a**) cystic proliferation with sebaceous differentiation (hematoxylin and eosin; ×40; ×100) and (**b**) masses of squamous and epithelial cells. Histopathology slides showed (**c**) a predominance of a cystic pattern over a solid one and (**d**) cystic space filled with a sebaceous material (hematoxylin and eosin; ×200).

Histologically, paraffin sections stained with hematoxylin and eosin showed a microcystic appearance with numerous sebaceous glands, with various dimensions, embedded in stromal fibrosis (Figure 3a,b). Occasionally, the glandular structures are dilated and contain sebaceous material (Figure 3c,d).

The patient’s postoperative course was uneventful; until now (4 months later), she has remained without evidence of recurrence. The patient is still under follow-up.

## 3. Discussion

Although most commonly associated with hair follicles, ectopic sebaceous glands can be identified independently. These lesions can be found mainly on the eyelids and oral mucosa [9]. Occasionally it may arise in the major salivary glands [10]. Benign tumors can rarely originate from these sebaceous glands, such as sebaceous adenoma, sebaceous lymphadenoma, or sebaceous carcinoma [7].

Despite the common occurrence of sebaceous differentiation in salivary glands [11], sebaceous adenomas are very rarely encountered in major salivary glands, accounting for 0.1 percent of all salivary gland tumors [7] and less than 0.5 percent of all salivary adenomas [12]. Most of them develop in the parotid gland, but they can rarely develop in the submandibular and minor salivary glands [8]. There was no impact on sebaceous adenomas of the parotid gland in either gender [13]. The majority of cases are diagnosed in patients 60 or older [11].

We performed a search on two databases, PubMed^®^ and Web Of Science^®^, published during the all-time topic, screening for the keyword “sebaceous adenoma of the parotid gland”.

After the screening, we found a few reports concerning sebaceous adenoma located in the parotid gland (Table 1).

Following the analysis of the all-time literature, the current case is the tenth reported case of sebaceous adenoma with localization in the parotid gland. The case presented showed differentiated sebaceous lobules accompanied by a fibrous stroma.

Thus, de Vicente-Rodríguez reported the histopathological appearance in a case of sebaceous adenoma located in the parotid gland [20]. Foote and Frazell reviewed 877 tumors of the major salivary glands and revealed only one case of sebaceous adenoma of the parotid gland [14]. Bab and Ulmansky reported a case of an adenoid cystic carcinoma associated with a sebaceous cell adenoma [15]. Amongst the 183 cases reported by Pieterse and Seymour, there was one case of sebaceous adenoma [16]. Gnepp and Brannon reviewed 21 cases of primary salivary gland sebaceous tumors and found three sebaceous adenomas located at the parotid gland [17]. Due to the low prevalence of the sebaceous adenomas of the parotid gland and the low number of published cases, obtaining an exhaustive description of the characteristics of the disease is difficult.

Sebaceous adenomas of the skin sites may be associated with nonpolyposis colorectal cancer, a condition known as Muir–Torre Syndrome [23,24], an autosomal-dominant disorder characterized by a combination of at least one cutaneous neoplasm and at least one visceral malignancy [25]. No such relationship between these tumors within the major salivary glands and Muir–Torre Syndrome has been documented [26].

Generally, sebaceous adenomas in the parotid gland are painless and slow-growing well, encapsulated lesions that do not infiltrate the surrounding normal tissues, with no facial nerve involvement. The tumor size typically ranges from 0.5 to 5 cm in diameter. They histologically appear as white-yellowish nodules that have a microcystic pattern, with abundant well, differentiated sebaceous glands of variable size [7,27]. The absence of the lymphoid component did not support a diagnosis of sebaceous lymphadenoma [20].

Besides clinical examination, ultrasonography (US), computed tomography (CT), and magnetic resonance imaging (MRI) are the most common radiological procedures for the diagnosis of tumor-like lesions of the salivary glands. According to Haynes et al., the initial diagnostic test for salivary gland tumors should be computed tomography (CT), which offers information regarding the mass’s size, extent, location, content, and consistency [28]. However, ultrasound (US) is accepted as the first imaging method for assessing soft-tissue diseases in the head and neck, including the major salivary glands, being beneficial in distinguishing cystic from solid lesions [29]. The US can be used to establish the need for imaging procedures, particularly in those lesions showing malignant features on ultrasonography or large masses located in the deep lobe [30]. Due to poor economic status, our patient was not willing for CT; hence, the US was conducted.

There is no consensus about using fine-needle aspiration biopsy (FNAB) in major salivary gland tumors [31]. According to some researchers, parotid tumors other than pleomorphic adenomas are uncommon, and pathologists may misdiagnose FNAB if they are not specialized in parotid tumors [32]. In the current case, no FNA was performed as that was a frankly benign parotid tumor, and the plan was to proceed for superficial parotidectomy directly.

Differential diagnosis should include other benign parotid tumors with solid mass with cystic change patterns [18].

Usually, these benign tumors can be removed, and they are not likely to recur if adequately excised. Since there is no evidence for efficacy, radiation therapy should not be considered an alternative to surgery in sebaceous adenoma. Thus, curative surgical excision is the treatment of choice.

## 4. Conclusions

The authors described an extremely rare case of sebaceous adenomas located in the parotid gland, which might help to enrich the clinical spectrum of this rare site. This case illustrates that sebaceous adenoma should be considered in the differential diagnosis of every soft tissue tumor in any major salivary gland, especially when it occurs in the parotid gland region.

## Figures and Tables

**Table 1:** Characteristics of sebaceous adenoma involving the parotid gland reported in previously published literature.

	Author (Year) [Ref]	Age (Years)	Sex	Clinical Features of the Tumor	Follow-Up
1	Foote and Frazell (1953) [14]	-	-	3.5 cm mass	-
2	Bab and Ulmansky (1979) [15]	57	F	-	dead at 6 months of adenoid cystic carcinoma, no recurrence
3	Pieters and Seymour (1981) [16]	-	-	-	-
4	Gnepp and Brannon (1984) [17]	71 74	F M	14 mm diameter mass 23 mm diameter mass	1.5 years, no recurrence died with no evidence of disease at 6 years
5	Shen (1994) [18]	39	M	5 × 6 × 4 cm mass	19 months, no recurrence
6	Derias (1994) [19]	73	F	-	-
7	de Vicente Rodríguez (2006) [20]	59	F	3.5 cm mass	5 years, no recurrence
8	Welch (2007) [21]	2	M	5 cm mass	-
9	Apple (2009) [22]	29	F	4 cm mass	-
10	Present case (2022)	65	F	3.2 × 2 × 1.6 cm mass	4 months, no recurrence

F—female; M—male.
